# PDMS Organ-On-Chip Design and Fabrication: Strategies for Improving Fluidic Integration and Chip Robustness of Rapidly Prototyped Microfluidic In Vitro Models

**DOI:** 10.3390/mi13101573

**Published:** 2022-09-22

**Authors:** Tiffany C. Cameron, Avineet Randhawa, Samantha M. Grist, Tanya Bennet, Jessica Hua, Luis G. Alde, Tara M. Caffrey, Cheryl L. Wellington, Karen C. Cheung

**Affiliations:** 1 School of Biomedical Engineering, University of British Columbia, Vancouver, BC V6T 1Z3, Canada; 2 Centre for Blood Research, University of British Columbia, Vancouver, BC V6T 1Z3, Canada; 3 Dream Photonics Inc., Vancouver, BC V6T 0A7, Canada; 4 Department of Chemistry, University of British Columbia, Vancouver, BC V6T 1Z1, Canada; 5 Department of Pathology and Laboratory Medicine, University of British Columbia, Vancouver, BC V6T 2B5, Canada; 6 Djavad Mowafaghian Centre for Brain Health, University of British Columbia, Vancouver, BC V6T 1Z3, Canada; 7 Department of Electrical and Computer Engineering, University of British Columbia, Vancouver, BC V6T 1Z4, Canada

**Keywords:** microfluidic, organ-on-chip, rapid prototyping, cell culture

## Abstract

The PDMS-based microfluidic organ-on-chip platform represents an exciting paradigm that has enjoyed a rapid rise in popularity and adoption. A particularly promising element of this platform is its amenability to rapid manufacturing strategies, which can enable quick adaptations through iterative prototyping. These strategies, however, come with challenges; fluid flow, for example, a core principle of organs-on-chip and the physiology they aim to model, necessitates robust, leak-free channels for potentially long (multi-week) culture durations. In this report, we describe microfluidic chip fabrication methods and strategies that are aimed at overcoming these difficulties; we employ a subset of these strategies to a blood–brain-barrier-on-chip, with others applied to a small-airway-on-chip. Design approaches are detailed with considerations presented for readers. Results pertaining to fabrication parameters we aimed to improve (e.g., the thickness uniformity of molded PDMS), as well as illustrative results pertaining to the establishment of cell cultures using these methods will also be presented.

## 1. Introduction

The use of microfluidic chips in biological research and drug discovery is an evolving technology that requires interdisciplinary innovation across the sciences and engineering. The PDMS microfluidic chip has enabled the development of human cellular models, commonly referred to as organs-on-chips, which replicate elements of in vivo physiology absent in traditional, static, well-plate based in vitro models [1]. They have the potential to better represent the in vivo condition, with applications ranging from drug screening to disease modeling [2,3]. Additionally, microfluidic systems can integrate a plethora of microsensors for continuous monitoring of multiple culture metrics—from excretion of soluble biomarkers to trans-epithelial electrical resistance [4,5]. The continued development and refinement of microfluidic systems is well positioned to make a valuable contribution to drug discovery.

Microfluidic chips for drug discovery applications often require multi-layer patterning, as well as functionality under a flow-based environment. In organ-on-chip applications, multi-layer patterning is useful for developing a more physiologically relevant cellular environment, such as a co-culture separated by a porous membrane [6,7,8]. In addition, the inclusion of fluid flow in the in vitro model allows for replicating the shear stress exerted by blood flow onto the cell layer or airflow over lung epithelia [9,10]. Traditionally, multi-layered microfluidic chips are manufactured in high throughput using hot embossing [11,12] or injection molding [13,14]. For lower throughput, research-based applications, soft lithography is often preferred, as it can leverage the low cost, biocompatible, and oxygen permeable heat-cured polymer polydimethylsiloxane (PDMS) [15,16]. Recently, a transition to forming master molds using rapid prototyping techniques has gained prominence [17,18], as the traditional method of photolithography is costly and requires a clean room facility—a factor partially responsible for the lower adoption levels PDMS has seen outside of academic environments [19]. PDMS can also easily form multi-layered patterns due to its ability to be irreversibly bonded to PDMS or to glass using air plasma, partial curing, or corona discharge bonding [20,21,22]. Commercially available microfluidic chips, on the other hand, include those made from glass, and polymers including polymethyl methacrylate, polystyrene, cyclic olefin copolymer, polycarbonate. After designs are optimized and are set for commercial production, thermoplastic polymeric chips have been manufactured using hot embossing or injection molding. Glass chips, while historically made from wet chemical etching or laser ablation [23], can also be made using newer, more rapid methods including ultrafast laser inscription [24] and glass imprinting [25].

The flow required by organ-on-chip models can be achieved passively, through gravity driven or capillary action, or actively, through an external pump. However, there remains challenges with connecting micro-sized channels with macro-sized commercially available connectors and tubing, commonly known as fluidic interconnects [26,27,28]. At these interfaces, creating leak-proof and durable connections is integral to the overall robustness of the chip during potentially long operation periods; the small-airway-on-chip reported by Benam et al., for example, was cultured for over three weeks [29]. There have been many attempts to overcome this challenge, such as using a compressing tubing within a microfluidic chip [30], using threaded inlets [31] or using adhesive-based strategies such as an epoxy to augment, for example, a pressure-fit PDMS-steel needle seal [32]. These methods are widely used, though contain some challenges. Using compression of tubing within the channel requires that the user does not dent the flexible PDMS channel, potentially forming a nucleus for bubble formation (detrimental to culture health and imaging capabilities). Additionally, threading inlets at the interface of a microfluidic chip can be cumbersome and become unwieldy when many inputs/outputs (I/O) are required. Lastly, epoxy can dissolve and delaminate from PDMS when exposed to alcohols used for disinfection such as ethanol; it, furthermore, has the potential to occlude the channel during hardening. There are many considerations when designing fluidic systems for organ-on-chip applications. For example, side-loaded ports for the fluid line may be desirable as they allow the microfluidic chip to be flipped for inverted imaging, especially useful for multi-compartment devices with multiple cell types. Side-loading, however, complicates the conventional technique of biopsy-punching to create fluidic interfaces. Compression of PDMS is commonly employed to augment fluidic seals and improve the robustness of fluidics; however, conventional fabrication methods often lead to non-uniformities in PDMS thickness, which have the potential to deform channels under said compression, undermining the original objective [33]. Additionally, some organ-on-chip applications require long-term culture durations; for example, the differentiation period of primary airway epithelia is on the order of weeks [34]. As such, protocols, and strategies for improving the ease of handling while minimizing contamination risk are essential.

In this work, we introduce key fabrication steps and strategies to improve both fabrication yield and device performance for applications in organs-on-chips development. These key steps include increasing the uniformity of PDMS thickness produced from master molds, employing a digital-light-processing 3D printer purpose-built for rapid production of PDMS master molds, as well as exploring methods for reinforcing PDMS bonding and fluidic integration. Selected aspects pertaining to the latter were applied to two separate organ-on-chip designs: an airway-on-chip model and a blood–brain barrier (BBB) model. Differences and advantages to the fabrication strategies are summarized in Table 1. We show how these rapid prototyping strategies can improve important organ-on-chip performance metrics such as incidents of leakage, confinement of culture growth to microfluidic channels, and overall success rate of producing morphologically typical cultures. We also characterize the surface finish obtained.

## 2. Materials and Methods

### 2.1. Fabrication and Design of the Master Molds

#### 2.1.1. Fabrication of the Master Molds

Master molds were fabricated using a digital light processing (DLP) printer (MiiCraft Ultra 50, Distributor: Creative CadWorks) or a stereolithography (SLA) printer (Form2, Formlabs). Mold design was completed via CAD (computer-assisted design) software (SOLIDWORKS Dassault Systèmes, Vélizy, France) and resulting parts were exported into file formats accepted by each 3D printer’s instrumentation software. Specifics pertaining to mold print settings and post-processing can be found in the Supplementary Materials.

The organ-on-chips consist of two layers of PDMS that are separated by a porous, transparent polyester membrane with a pore size of 0.4 µm, a porosity of 2.0 × 10^6^ cm^2^ and a thickness of 12 µm (it4ip, cat. No. 200M12/620N403/47). This structure of microfluidic chip allows for potential to include cell-to-cell interactions as well as the ability to apply physiological shear stress on the cell layer. Two organ-on-chips designs are used, a blood–brain barrier (BBB) chip and an airway-on-chip.

##### Design of the BBB Chip

**Figure 1:**
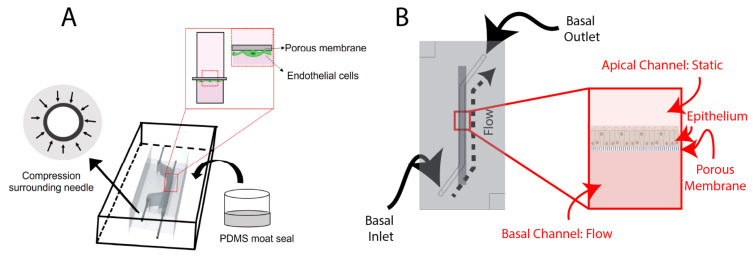
Schematic overviews of: (**A**) the BBB-on-chip; and (**B**) the airway-on-chip. Key features of the BBB-on-chip include making molds of higher gauge (smaller outer diameter) needles before adding the I/O needles and sealing the chip using a PDMS moat. Both chips incorporate a porous membrane to separate the channels, with the BBB-on-chip seeding endothelial cells basally and the airway-on-chip seeding epithelial cells apically. Portions of this figure were created using https://biorender.com (accessed on 20 August 2022).

The BBB chip (Figure 1A) includes a static top reservoir of 1 mm width, 18 mm length (straight), (5 mm slanted), and 5 mm height. The inlet and outlets (I/O) are inserted from the side of the PDMS. Before casting the PDMS, 25G I/O needles McMaster-Carr) are manually inserted into the mold to sacrificially mold fluidic port features. Employing needles that are of a higher gauge (lower external diameter) than the needle that is intended to be used as a fluidic interconnect creates compression surrounding the needle, improving fluidic seal integrity. Finally, after removing the 25G needles, ½” 22G straight needles (McMaster-Carr) were inserted into the molded fluidic ports for the top and bottom channels.

##### Design of the Airway-On-Chip

The airway-on-chip (Figure 1B) channel measures 1.3 mm wide, 0.75 mm high, and 25 mm long; these dimensions were chosen to achieve overall culture areas equivalent to commonly used 24-well Transwell^®^ inserts while maintaining a hydraulic diameter under 1 mm to better replicate small airway physiology [36,37].

The airway-on-chip is composed of a monolayer of airway epithelia; as such, it does not benefit from the ability for dual-side imaging (or, flipping the chip under a microscope) enabled by side-loaded fluidics. As such, it employed top-loaded fluidic ports, which are partially defined by raised columnar features in the top-layer master mold and hollowing is completed using a tissue biopsy punch (TedPella, 0.5 mm). Importantly, both the diameter of these features in the master mold as well as the diameter of the biopsy punch is undersized, relative to the 22G needle which is pressure-fit into the PDMS. Additional considerations regarding fluidic port design and integration are provided in the Supplementary Materials.

The mold edge heights define the thickness of the PDMS, which is 6 mm; this value, while large relative to other microfluidic organs-on-chip, was found to impart greater stability to the stainless-steel needles used to interface fluidics. Alignment features, consisting of recessed and protruding squares on the top and bottom chip halves with a 100 µm margin, are also included at two corners to assist with manual alignment during fabrication.

### 2.2. Fabrication and Assembly of the Microfluidic Chips

#### 2.2.1. PDMS Preparation and Bonding

**Figure 2:**
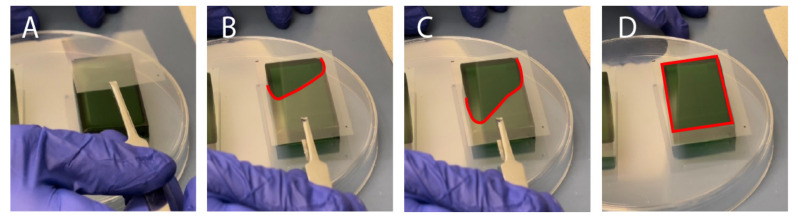
Slowly and carefully placing the transparency film over the uncured, PDMS-filled mold minimizes formation of bubbles: (**A**–**D**) depict the different stages of slowly lowering the transparency onto the convex PDMS surface, starting from the top left corner. A red dotted outline is used to depict the area of the transparency film that is in contact with the PDMS.

A 10:1 mixture of PDMS elastomer base and curing agent (Dow Sylgard^®^ 184 Silicon Elastomer) was mixed in a planetary centrifugal mixer (Thinky, cat. No. ARE 310) for 90 s at 2000 RPM followed by 60 s at 2200 RPM. The PDMS mixture was casted into the molds and placed into a vacuum chamber for removal of air bubbles in the PDMS mixture for 30–60 min. Following degassing, a transparency film (Apollo, purchased from Amazon.ca (accessed on 9 December 2021)), cut to fit the mold’s footprint with an excess 1–2 cm border, was placed over top of the mold. In order to minimize the formation of bubbles (or air-pockets) forming at the transparency-PDMS surface, a tweezer was used to enable gradual laying of the film from one side to the other of the mold (Figure 2A–D). After placing the transparency, a flat piece of 3/32” thick acrylic was placed on top of the film to enable even compression by an aluminum weight (total weight 200–300 g). The molds were transferred to an oven (65 °C) to cure for at least four hours. Then, the PDMS layers were removed from the molds using an Exacto-knife, scalpel, or razor blade and excess debris were removed from the surfaces using Scotch™ Magic tape and compressed air.

To identify the bonding performance of the PDMS-PDMS or PDMS-glass bonds, first, a visual inspection was made, to ensure all areas of the chip were transparent, then a leak-test was performed by manually pushing food-colored dyed PBS through tubing attached to the chip.

##### Oxygen Plasma Bonding—BBB Chip

For the BBB organ-on-chip application, using tweezers, a membrane (it4ip, cat. No. 200M12/620N403/47) was carefully set into position to cover the channel. The two layers of PDMS were treated with air plasma (Harrick plasma cleaner) at ~600 mTorr for 1 min before manually aligning the channels and compressing the PDMS layers together for approximately 30 s. The plasma-bonded PDMS layers were then transferred to a 65 °C oven for at least 2 h to complete the bonding procedure.

##### Partial Curing—Airway-On-Chip

The airway-on-chip leveraged PDMS-to-PDMS bonding through partial curing, as characterized by Eddings et al. [22]. This process comprised an initial, partial cure of PDMS-filled molds for 60 min at 65 °C following which molds were removed from the oven for membrane alignment, punching, and conjoining the two chip halves. The chips were then covered with Scotch™ tape and returned to the oven under compression for an additional three hours to allow polymerization to complete, yielding a bonded and complete microfluidic device.

#### 2.2.2. Fluidic Integration and Reinforcement

##### BBB Chip

**Figure 3:**
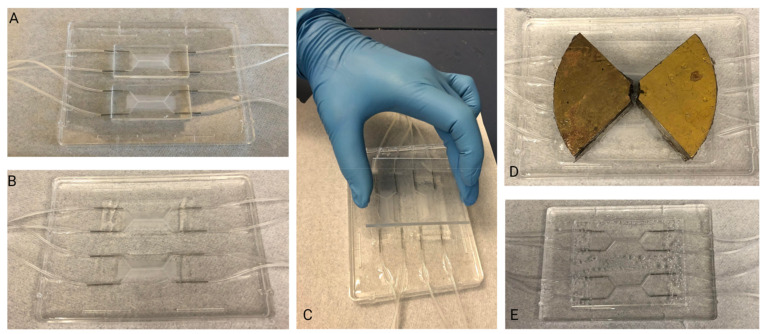
PDMS Moat Fabrication Steps: (**A**) Assembled chips are placed into a flat, smooth bottom dish. (**B**) PDMS is poured into the dish ensuring that the chips are completely surrounded. (**C**) An acrylic sheet is placed on top of the chips to ensure the area of interest of the chip (central channel) remains clear of PDMS. (**D**) A weight is used to weigh the chips down within the liquid PDMS. (**E**) Once cured, PDMS block is removed from dish. The chips are now embedded in PDMS block and able to be immediately connected to fluidics system.

In order to augment the fluidic interface in the side-loading BBB chip, a PDMS encasement (“moat”) (Figure 3) was prepared. This encasement was created by taping the tubing in position, then using a flat, smooth bottom dish into which chips were set. To preserve PDMS, the chips were placed side-by-side and tape was used to keep them together. Then, liquid PDMS was poured around the chips, such that the PDMS surrounded the inlet and outlet tubing and fully encased the system. Additionally, a weight was placed on top of the chips to keep them on the bottom of the dish. Finally, the moat was cured at 65 °C overnight.

##### Airway-On-Chip

To improve the robustness of fluidic interfaces and operation of the airway-on-chip over long (≥10 day) culture durations, two approaches were explored and ultimately included. These consist of: (1) mechanical reinforcement of the reversible PDMS-to-PET-membrane bond through a rigid plastic clamp and (2) fluidic seal enhancement via dispensing of uncured PDMS around fluidic interfaces.

The airway-on-chip model features cells cultured on a PET membrane that is sandwiched between two PDMS microfluidic channels. If the PDMS is not bonded well and there is a gap between the PDMS and the membrane, cells can grow into the thin gap. This can lead to unpredictable culture sizes and cell numbers, which may undermine reproducibility from chip to chip. Mechanical reinforcement of the reversible PDMS-to-PET-membrane bond to restrict cell-growth to the microfluidic channel was found to be necessary and achieved through an acrylic clamp. Quarter-inch cast acrylic (McMaster-Carr, Elmhurst, IL, USA) was purchased in 12″-by-12″ sheets and cut with a VLS 2.30 (Universal Laser Systems, Scottsdale, AZ, USA) CO_2_ laser cutter; outer dimensions of the clamp measured 73 mm-by-48 mm, 20 mm larger than the chip along both the length and width. A square cutout (4 mm-by-4 mm) was centered over each fluidic inlet/outlet port to enable access, and two circular cut-outs sized for 8–32 threaded rods (McMaster-Carr) were made collinear with the top channel. Compression was achieved utilizing 8–32 thumb nuts (McMaster-Carr) coupled with washers for improved uniformity of force distribution. Following assembly of the chip-clamp unit, 22-gauge stainless steel needles (McMaster-Carr) with a 1″ 90-degree bend were inserted into the fluidic access ports with the other end of the needle coupled to a 10 cm length of 0.02″ ID microbore Tygon tubing (VWR). The other end of this tubing length was coupled to a 0.5″ 22GA straight dispensing tip with a luer-lock connection for ease of fluidic interfacing and handling. A strip of tape was placed on the clamp, overtop of the fluidic lines for added stability during handling. Liquid PDMS was, then, dispensed via pipette (with the tip scissor-cut to increase the opening size, due to the fluid’s viscosity) into the clamp’s cut-outs to augment the compression fit seal between the needles and the PDMS, and cured at room temperature for 48 h. The thickness of the clamp as well as the PDMS of the top chip layer is sized such that the bent stainless steel needle rests on the acrylic when the bent portion penetrates approximately 3.5 mm into the 5.25 mm thick (with the channel depth subtracted from the overall layer thickness) PDMS (not too deep so as to risk piercing the PDMS of the bottom layer while retaining a stable pressure-fit seal).

### 2.3. Microfluidic Cell Culture

#### 2.3.1. BBB Chip

For the BBB chip, human brain microvascular endothelial cells (HBMECs) (Lonza, Basel, Switzerland, lot# 376.01.03.01.2F) were thawed from passage 6 and used between passage 6–9. HBMECs were cultured onto fibronectin-coated plates and passaged at 80% confluency. Cells were lifted using 0.05% trypsin and cultured with EGM-2 bullet kit containing 2% *v/v* FBS (Lonza, cat. no. CC-3162).

A 1 mL syringe with a 22G dispensing tip was inserted into chip tubing to perform all liquid exchanges. For the BBB chip, a 10 min 70% ethanol rinse, followed by two PBS washes, and an overnight equilibration using EGM-2 media was used to prepare the chips for ECM-coating. For the airway-on-chip, a 20 min ethanol incubation preceded coating.

For the BBB chip, a coating of 0.4 mg/mL collagen IV (Sigma Aldrich, St. Louis, MO, USA, cat. no. c5533), 0.1 mg/mL fibronectin (Sigma Aldrich, cat. no. F1141), and 0.1 mg/mL laminin (Sigma Aldrich, cat. no. L2020) was applied the day after equilibration and left overnight in the fridge. Lastly, the chip was washed twice with PBS. Then, HBMECs were seeded into the chip at a density of 5 million cells/mL and then attached to a pump for at least 5 days to allow for perfusion through the endothelial channel. A syringe pump was used to provide media perfusion resulting in shear stress values of ~0.001 dyne/cm^2^ within the channel. The top channel media was exchanged every two days. The profile of the flow rate delivered by the syringe pump was measured using microfluidic flow sensors, as described in the Supplementary Materials.

#### 2.3.2. Airway-On-Chip

Three vials of Calu-3 human lung adenocarcinoma airway epithelial cell line were obtained from ATCC and expanded and maintained in T-75 flasks using Eagle’s Minimum Essential Medium (Corning, Corning, NY, USA) supplemented with fetal bovine serum (MilliporeSigma, St. Louis, MO, USA, Cat: F1051, Lot: 19D019), and Antibiotic-Antimycotic (Thermo, Waltham, MA, USA, Cat: 15240-062, Lot: 2441423). Cells were passaged at 80% confluence and used for experiments between Passages 20–25.

Microfluidic chips were filled with a 100μg/mL rat-tail-collagen (Corning, Cat: 354236, Lot: 1049001) solution in PBS and incubated in a biosafety cabinet overnight to complete membrane coating. Following flushing and equilibration with complete culture medium, chips were seeded via syringe-infusion with a suspension containing six-million cells per milliliter. After the suspension was loaded, luer caps (McMaster-Carr) were coupled to each fluidic line and the chips were transferred to an incubator to allow cells to attach for five hours.

Following the five-hour attachment period, luer caps were removed from the basal channel inlet and outlet lines. They were, then, connected to a recirculating flow system, wherein a peristaltic pump was positioned to pull culture medium through the basal channel and into a 2 mL medium reservoir containing the same medium used for culture maintenance. The recirculating flow regime enables the application of physiologically relevant shear stresses while minimizing reagent consumption. Reservoir culture medium was contained in five milliliter screw-cap MacroTubes™ (FroggaBio, Toronto, ON, Canada); their caps were punctured with 16-gauge needles (Becton, Dickinson and Company, Franklin Lakes, NJ, USA), creating an opening just large enough to accommodate the insertion of the microfluidic tubing employed. The tubing expands in the warmer environment of the CO_2_ cell culture incubator, creating a tighter seal for the duration of the experiment. The following day, the apical channel was washed with fresh complete medium, and maintained in a submerged state for the duration of the experiment.

The reservoir volume was chosen based on manufacturer’s recommendations for Transwell^®^ 24-well culture inserts; this recommendation stipulates a culture volume of 600 µL to support a culture area of 0.33 square centimeters, equivalent to the airway-on-chip. Exchange of medium in a Transwell plate every two days is a common culture regime that has been demonstrated to produce functional cultures [38,39]. Direct communication with ATCC yielded an upper bound on culture medium stability in a cell-culture incubator of four days, and this exchange interval has been employed in previously reported organ-on-chip cultures; as such, the 600 µL value was doubled to 1.2 mL and a factor of safety was added to arrive at the 2 mL reservoir volume, with reservoir exchanges and apical channel washes taking place every three to four days [40]. The initial flow rate employed was 300 μL/min, which was ramped up following the second media reservoir exchange and apical channel wash to 540 μL/min, corresponding to a wall shear stress between 0.6 and 0.7 dyne per square centimeter exerted basally at the higher flow rate—small airway epithelia experience a range of 0.5–3 dynes per square centimeter at rest due to airflow in vivo [10,41].

Cultures were maintained for eleven days, at which point Calu-3 epithelia cultured on Transwell inserts reach peak barrier integrity (measured based on apparent permeability to fluorescein isothiocyanate-conjugated dextrans) [39].

### 2.4. Cell Culture Analysis

#### 2.4.1. BBB Chip

HBMECs were stained to visualize f-actin and nuclei. All staining was performed in-chip. A 1 mL syringe with a 22G dispensing tip was inserted into the chip tubing to perform all liquid exchanges. For f-actin staining, samples were fixed, with 4% paraformaldehyde in PBS and left at room temperature for 15 min. Samples were washed twice with PBS for 10 min. After washing the fixative 2 × 5 min with PBS, samples were incubated with the 66 µM dimethyl sulfoxide (DMSO) Alexa-Fluor-488 Phalloidin stock solution at a 1:400 dilution in PBS for 1 h at room temperature. Samples were left in a dark, covered container to prevent photobleaching and evaporation while staining. Then, samples are washed 2 × 5 min with PBS (1×). For nuclei staining, cells were either stained live or fixed, with Hoechst (Thermofisher Scientific, Waltham, MA, USA, cat. no. H3570) at a 1:1000 dilution in EGM-2 or PBS. Live cells were washed with EGM-2 and fixed cells were washed 2 × 5 min with PBS (1×).

BBB chips were disassembled using an Exacto knife and pliers, and the membrane was removed and placed onto a glass slide with tweezers. The membrane was placed with the cell side facing upwards. ProLong^®^ gold Anti-Fade containing 4′,6-diamidino-2-phenylindole (DAPI) was used to mount the membrane and a cover slip was placed onto the membrane; because the cells were stained with Hoechst, the DAPI was not necessary but was included in the commercial formulation. The edges of the coverslip were sealed using clear nail polish to prevent evaporation. The mounted samples were stored at 4 °C.

#### 2.4.2. Airway-On-Chip

On Day 11, the airways-on-chip were fixed in situ for preservation and staining. All steps involving manual syringe-infusion of chips are conducted separately for each channel, while the other is occluded; for example, when washing with PBS, the apical channel is washed first while the basal channel is closed with luer-caps. Infusion of a channel is confirmed both visually in the channel, for example by tracking small bubbles that may be introduced during injection, as well as via confirmation of three droplets forming at the outlet of the channel being infused (chosen conservatively, based on the internal volume of the channel). The single-channel infusion with the second channel blocked minimized the chance of crossflow, which is especially important earlier in the experiment when cells have not adhered or formed a barrier tighter than the porous membrane itself.

Chips were washed with PBS three times; following this, a 4% methanol-free formaldehyde solution in PBS (Thermofisher, Pierce) was infused to fill each channel. This solution was incubated for 15 min at room temperature, following which an additional three PBS washes were performed. Cells were then permeabilized by injection of a 0.02% Tween-20 solution in PBS, which was incubated for 15 min followed by an additional three PBS washes. Chips were then blocked with a 1% BSA solution for 90 min before loading the working concentration of Alexa-Fluor 488 Phalloidin (Thermofisher) stain (165 nM in PBS containing 1% BSA), which was incubated for 45 min. Chips were, then, washed a final three times before fluidic and clamp disassembly.

Four incisions along each edge of the membrane, through the PDMS, were made to extract the cell-laden membrane from the chip. Extracted membranes were promptly laid onto a microscope slide with two drops of ProLong Gold antifade reagent with DAPI (Thermofisher); a coverslip was carefully placed atop the membrane and the mounting media was cured under a coverslip at room temperature in the dark overnight. The following morning, slides were transferred to a slide-box for storage at 4 °C.

### 2.5. Imaging

Images of HBMECs were taken on an inverted microscope (Zeiss, Observer.Z1, Oberkochen, Germany). Images of Calu-3s were taken on an inverted microscope with epifluorescent capabilities and intermediate magnification switching from 1.0× to 1.5× (Nikon Eclipse Ti2-E (Nikon, Tokyo, Japan)); 10×/NA 0.3, dry (Nikon: MRH10105) and 40×/NA 0.6, dry (Nikon: MRH48430) objectives were used in conjunction with 432 nm (Semrock, Rochester, NY, USA, 36 nm bandwidth, cat: FF01-432/36) and 515 nm (Semrock, 30 nm bandwidth, cat: FF01-515/30) single bandpass emission filters for DAPI and Alexa-Fluor-488-Phalloidin microscopy, respectively).

### 2.6. PDMS Thickness Uniformity Characterisation

**Figure 4:**
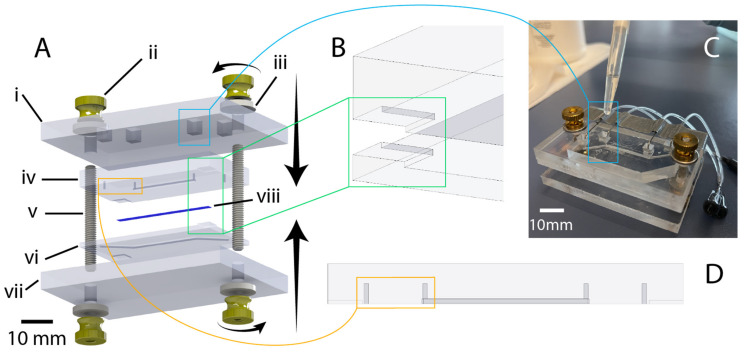
Illustration of chip design and highlighting of critical features. (**A**) exploded view of chip components (created using SolidWorks): i: 1/4-inch-thick acrylic top clamp, ii: 8–32 brass flanged thumb nut, iii: 8–32 oversized washer, iv: PDMS chip (top half), v: 8–32 threaded rod (1.5” length), vi: PDMS chip (bottom half), vii: 1/4-inch thick acrylic bottom clamp, viii: PET membrane. (**B**) illustration of corner alignment features to assist manual mating of chip halves during bonding. (**C**) Illustration of liquid-PDMS reinforcement of fluidic port seal via pipetting of uncured PDMS into top-clamp cutouts. (**D**) Highlighting of punch guides to assist in the accurate positioning and clear formation of manually punched through-holes for fluidic connection.

In microfluidic devices employing pressure clamps to aid in sealing (such as that depicted in Figure 4), the thickness uniformity of the PDMS critically impacts both the quality of the seal as well as channel integrity. Thickness nonuniformity can lead to uneven compression across the device, in turn leading to leakage in regions of low compression or channel deformation in regions of higher compression. We introduced the use of transparency films during curing to improve thickness uniformity and thus device performance. In order to characterize the impact of employing transparency films during PDMS curing, a separate PDMS microfluidic device with two, smaller, microfluidic channels was fabricated that would better highlight channel integrity under compression. Master molds were created on the same printers as for the organ-on-chip devices and fabrication methods were identical to the organs-on-chips. Three devices were fabricated without the use of a transparency film to quantify its effects on the flatness of a cured device, and three devices were fabricated with a transparency film for comparison.

Following fabrication, the PDMS devices were measured with calipers at the same five points equally spaced along the long edge of the device. The devices were then compressed against a silicon wafer and visualized under a brightfield epi-illumination microscope (Aven MicroVue (Aven, Ann Arbor, MI, USA)) to assess the deformation of channels. Compression was achieved using 3–32 bolts and a rigid, laser-cut, cast-acrylic washer; the same individual manually screwed the bolts using fingertips to achieve comparable compression across all tested devices.

### 2.7. PDMS and Mold Surface Roughness Characterisation

The surface topography and surface roughness of the mold interior and the subsequent PDMS gasket was investigated with an atomic force microscope (AFM). The images of three random spots on each respective surface were obtained using a Nanosurf EasyScan 2 AFM system (Nanosurf, Liestal, Switzerland) in tapping mode with Si tips over the three regions of area 10 µm × 10 µm with a 512 × 512 pixel resolution. The root mean square (RMS) was then calculated to quantify surface roughness.

## 3. Results

### 3.1. Surface Roughness of 3D Printed Molds

**Figure 5:**
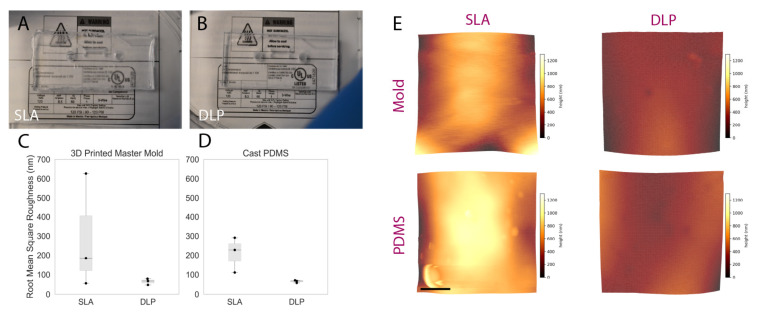
Comparing the performance of rapidly prototyped master molds. (**A**,**B**) comparison images captured of PDMS devices superimposed upon fine-point text to highlight optical clarity: (**A**) device cast from Form2-SLA-printed mold; (**B**) device cast from MiiCraft-DLP-printed mold. The device cast from the DLP-printed mold shows improved optical clarity. (**C**,**D**) boxplot summarizing RMS surface roughness of SLA and DLP molds alongside corresponding cast PDMS. Individual data points are represented as black dots superimposed on a boxplot, where the box height represents the interquartile range, the whiskers the total range, and the central line the median value. While statistical significance was not achieved, a trend towards lower roughness associated with DLP-printed molds and corresponding cast PDMS was exhibited—manifesting through the improved optical clarity illustrated over Subfigures (**A**,**B**). (**E**) representative topographic images obtained through AFM characterization of DLP and SLA 3D printed master molds (top) and cast PDMS (bottom); color scale corresponds *z*-axis height (surface profile), illustrating a more uniform surface obtained on the DLP-printed mold and cast PDMS. Scale bar represents 2.5 µm.

A visual comparison between the PDMS casted in both SLA and DLP molds is shown in Figure 5A,B, while surface roughness characterization is presented in Figure 5C,D. The manufacturer’s stated x-y as well as z resolutions of the DLP printer (MiiCraft Ultra 50) are 30 µm and 5–500 µm, respectively; corresponding values for the SLA printer (Formlabs Form2)—x-y and z resolution—are 140 µm and 25–100 µm. The z-resolution is user-selectable, and the values employed during mold fabrication were, for the DLP and SLA, 30 and 25 µm, respectively. The DLP printer used in this work was made specifically for PDMS casting, as the PDMS master mold resin is made with methacrylated monomers and oligomers and the light engine is optimized for this resin, allowing for an improved surface finish and minimal contact inhibition of PDMS curing (common in other 3D printing resins due to photoinitiators present in those resins) [42]. The resin in a DLP printer is cured layer-by-layer using a light projector and not point-by-point using a laser beam as in a SLA printer. We hypothesized that the difference in resolution between the two printers, as well as the layer-by-layer curing process, may be expected to contribute to reduced roughness in the printed molds. Figure 5A,B, illustrated that a PDMS microfluidic device casted on the DLP printer exhibits improved optical clarity compared to the PDMS microdevice that was casted using an SLA mold, likely due to the decreased scattering resulting from lower surface roughness of the DLP-printed mold. Measured surface roughness, correspondingly, was generally lower on the DLP-printed mold. Average RMS (root mean squared) surface roughness (± standard deviation over three areas measured) achieved using the DLP printer for the mold and casted PDMS were 64.7 ± 15.8 and 65.3 ± 6.7 nm, respectively. When using the SLA printer, mold and casted PDMS RMS surface roughness were 288.9 ± 298.9 and 209.63 ± 91.5 nm, respectively. A one-sided Mann–Whitney U test was performed to compare RMS roughness across both the DLP and SLA molds as well as the PDMS cast therein; the *p*-value associated with the comparison of molds was 0.2, while for the cast PDMS *p* = 0.05. Although these results did not indicate statistical significance over the presented measurements, this might be attributable to insufficient sample size (particularly impactful in non-parametric statistical tests), as we qualitatively observed less leakage and improved optical transparency with PDMS fabricated using the DLP printer.

### 3.2. PDMS Thickness

**Figure 6:**
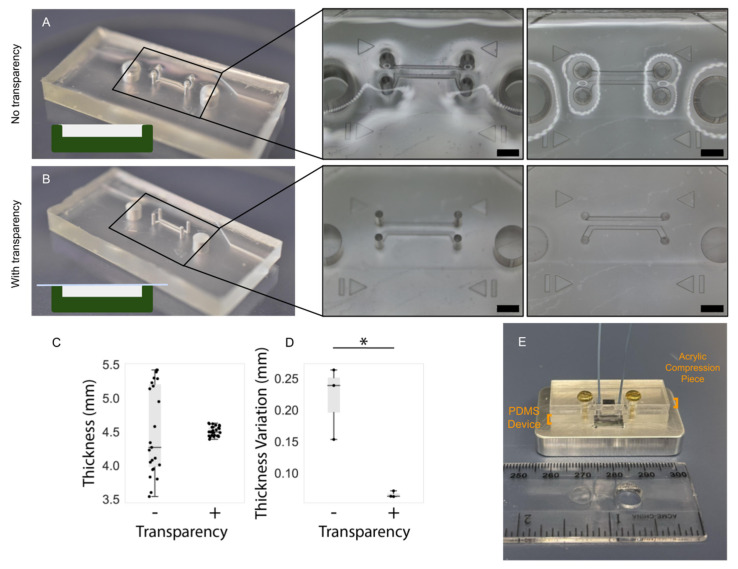
PDMS device fabrication with the transparency film method improves the flatness and uniformity of the top PDMS surface. Comparison of uniformity in PDMS thickness: (**A**) in absence of transparency film and; (**B**) when employing transparency film method, scale bar (black) represents 2 mm. Insets depict warping of the surface of the PDMS device fabricated without the transparency method, in regions surrounding the through-hole structures in the mold (visible as reflections and optical aberrations). In contrast, the surface of the device fabricated using transparencies is flat and does not impart these optical aberrations. (**C**) Comparing the impact of the transparency film on the measured thickness at several points on replicate PDMS devices and; (**D**) on the intra-device variability in PDMS thickness measurements taken at different regions on PDMS devices. The 15 raw thickness measurements for each set of devices is plotted in (**C**) and the standard deviation of the five measurements corresponding to each device is plotted in (**D**) (the asterisk denotes statistical significance at the 5% level). Individual data points are represented as black dots superimposed on a boxplot, where the box height represents the interquartile range, the whiskers the total range, and the central line the median value. (**E**) Illustration describing application of PDMS: microchannels molded into PDMS are sealed against a silicon wafer device to be subjected to flow via compression of a rigid, laser-cut, acrylic piece where non-uniformities in PDMS thickness may undermine the PDMS seal and flow path integrity.

Manual pouring of PDMS into master molds will generally result either in a slight overfilling or underfilling of the mold. The former manifests as a dome-like concave profile when viewed from the side; the latter results in the opposite as well as particularly stark height non-uniformity around features within the mold, especially when feature height is comparable to the depth of the mold. Furthermore, if the surface upon which the mold is resting during curing is not flat, a slant along the surface of the cured PDMS will develop corresponding to the angle of the curing surface. In order to overcome these effects (related to surface tension and/or surface angles), commercially available copier transparency film was used to provide a temporary lid (under an aluminum weight) to an overfilled mold that could be peeled off following curing to reveal a microfluidic device with more uniform thickness. Figure 6 highlights the thickness artefacts near features as well as their mitigation using the transparency film; thickness characterization of three devices fabricated with and without transparency films yielded a statistically significant increase in uniformity across the length of microfluidic devices fabricated with a transparency film (depicted graphically in Figure 6D).

**Figure 7:**
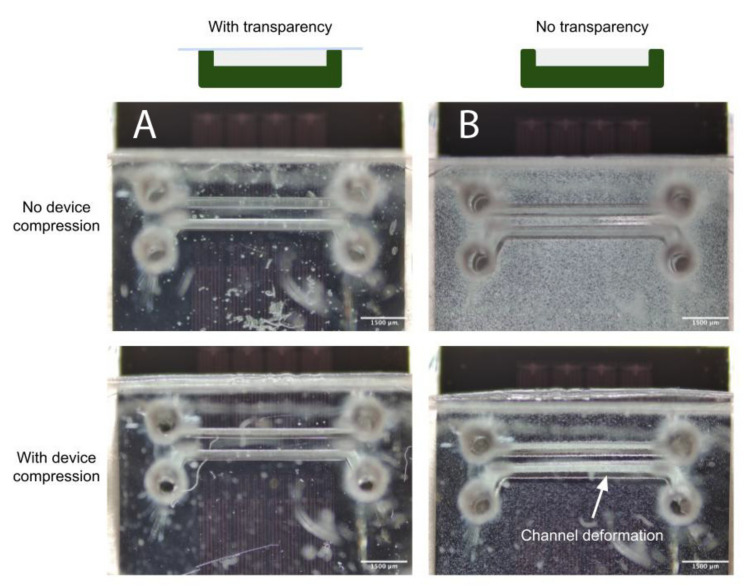
(**A**) When employing the transparency film during casting, the improved PDMS thickness uniformity facilitates straightforward reversible PDMS bonding using a compression setup; (**B**) Upon compression of the device against a flat surface to enclose the channels, increased channel deformation is evident under comparable force when employing a device fabricated without a transparency film.

Concerns surrounding non-uniformities in PDMS microdevice thickness include the increased likelihood of channel deformation under compression, commonly employed to augment fluidic seals and integrity for organ-on-chip applications (as discussed in the Airway-on-Chip subsection of Section 2.2.2, pertaining to clamp design) [33]. Figure 7 illustrates this phenomenon; channels in microfluidic devices fabricated without a transparency (less uniform in thickness) exhibit greater deformation under comparable compressive load. In quantifying the flatness uniformity, we observe much larger inter- and intra-device variability for devices fabricated without transparency than those fabricated with our transparency method (quantified interquartile range of 125 µm with transparency, and 1.225 mm without in Figure 6C; interquartile range of 4 µm with transparency, and 55 µm without in Figure 6D.

### 3.3. PDMS Encasement for Leakage Prevention: Comparison with Traditional Epoxy

We have determined that a combination of a compression-fit port, made using a higher gauge needle in the PDMS molding process, with a surrounding PDMS fortification or moat serves as a simple and effective method for connecting tubing to PDMS chips. Utilizing a PDMS moat-based method to reinforce fluidic connections rather than clamping or epoxy provides more flexibility within the device design. When combined with side-loaded ports, a PDMS sealing method can increase the devices compatibility with needle based sacrificial molding techniques used for creating 3D lumens with circular geometries.

**Figure 8:**
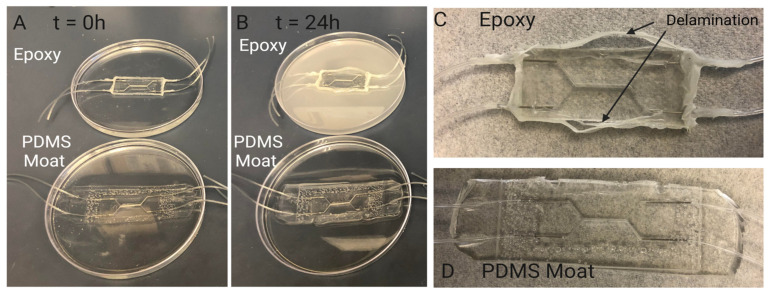
Investigating PDMS device sealing method. Sealing with PDMS moat prevents delamination during cell culture period. Comparison of degradation of sealing methods over 24 h: (**A**) Samples are submerged in 70% ethanol to accelerate the degradation seen in other aqueous liquids such as cell culture media. When initially placed in ethanol there are no visual signs of degradation in chips sealed with epoxy and chips sealed with a PDMS moat. (**B**) After 24 h, the ethanol in which the chip seal with epoxy is submerged exhibits a discoloration indicating a degradation of the epoxy. The chip sealed with PDMS moat shows no signs of discoloration or breakdown. Visual inspection of chips sealed with (**C**) epoxy and (**D**) PDMS moat (**D**) after 24hrs of submersion in ethanol. Over 24 h of submersion, the Epoxy-PDMS interface begins to delaminate and separation between the two layers can be seen macroscopically. This separation results in limited support for preventing leakage in chips when higher pressures are experienced within the device.

A common method to reinforce and secure ports/fluidic connections on PDMS microdevices includes utilizing epoxy to fix and seal tubing and/or needles in place [43]. The epoxy is used to seal any opening to prevent leakage and to prevent fluidic ports from disconnecting from the system during perfusion. However, for long term cell culture, epoxy can degrade over time resulting in delamination and separation between the epoxy-PDMS interface, as seen in Figure 8. An accelerated version of this separation was demonstrated by submerging chips sealed with epoxy or PDMS moats in 70% ethanol for 24 h. After 24 h, devices reinforced with epoxy showed signs of delamination and degradation while those reinforced with a PDMS moat did not. This indicates that for longer term cell culture utilizing a PDMS moat style of reinforcement may be more suitable for leakage prevention.

### 3.4. Success Rate of BBB Chips

BBB chips were seeded with endothelial cells and perfused with media for five days, and their performance was evaluated at different checkpoints. To assess the microfluidic chip performance, an initial checkpoint (Checkpoint 1) was used to identify if the PDMS-PDMS bonding was sufficient. To evaluate this checkpoint, a visual inspection was made after the bonding of the PDMS layers to ensure they were transparent, and also a manual leak test was performed. A second checkpoint (Checkpoint 2) was used to identify if any chips had leaked after being perfused with media for five days. This checkpoint was evaluated by visual inspection to ensure no tubing had disconnected or any leaks occurred due to insufficient bonding of PDMS or disconnected fluidic interconnects. Between checkpoints 1–2, there were 88% of 17 chips that had been viable after five days on pump. In this case, the failure mode was due to a tubing line becoming disconnected from the dispensing needle that contained a syringe full of media.

As some chips had endothelial cells seeded on Day 0 of the experiment, an additional checkpoint (Checkpoint 3) was used to identify if any cells attached to the membrane. This checkpoint can have many common failure modes, as it coincides with the cell layer optimization process. These failure modes could include cell detachment due to an uneven extracellular matrix layer, an air bubble trapped in the channel, or even insufficient media supply. Lastly, a final checkpoint (Checkpoint 4) was used to assess if there was a confluent monolayer of endothelial cells present. This was assessed with a visual inspection, and it should be noted that future optimization could be needed to ensure the monolayers present can be used in functional assays, such as a permeability assay. In this case, since only approximately half of the chips were seeded with cells (and the other half acted as controls), out of the eight chips seeded with endothelial cells, 57% formed a confluent monolayer, based on visual assessment. Table 2 outlines the checkpoints and their respective common and suspected failure modes.

### 3.5. Cell Proliferation and Morphology

**Figure 9:**
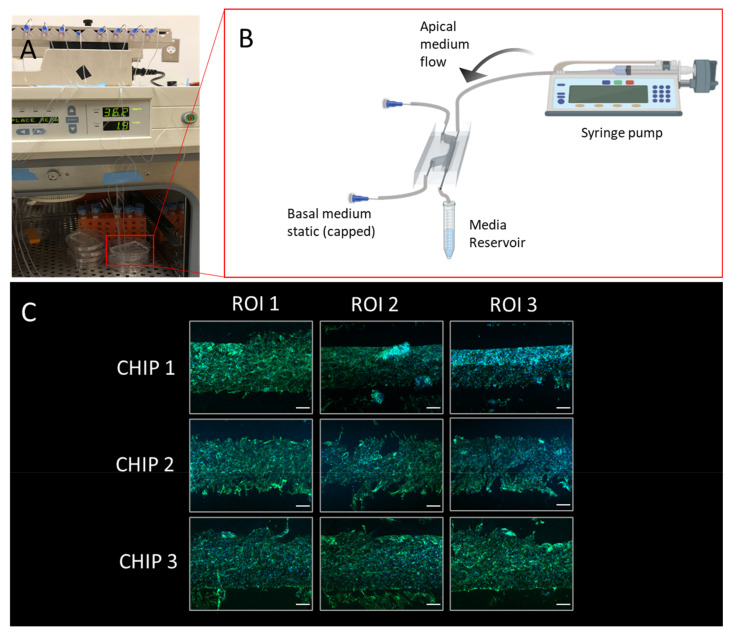
Illustration of blood–brain barrier chip cell culturing protocol and representative results: (**A**) Configuration with five chips in a cell culture incubator with medium reservoirs and pumping set-up; (**B**) Schematic illustrating culture environment of single chip, with apical channel static and syringe pump flow through basal channel of the BBB chip, created with BioRender.com; (**C**) Stitched microscopy image comprised of images capturing three regions of interest (ROIs) of the membrane post extraction and immunofluorescence staining (scale bar: 200 um), the green stain is phalloidin, and the blue stain is Hoechst 33342.

The BBB chip was seeded with HBMECs and perfused with media using a syringe pump for five days. As demonstrated in Figure 9, the cells adhered and remained on the membrane throughout the culture period. Based on visual assessment, the three chips shown produced a confluent HBMEC monolayer. The fabrication methods used with this BBB chip demonstrate the potential for a confluent monolayer to be formed and further optimized to be able to be used in functional assays. A common functional assay that is suitable for use with this chip is a permeability assay, which often relies on fluorescently labeled dextran being perfused through the channel of the chip, and sampled at different time points within the same (bottom) channel and the top channel [44,45]. A functional assay allows the user to assess the quality of the microtissue by obtaining a baseline measurement before adding any therapeutic or damaging treatments.

**Figure 10:**
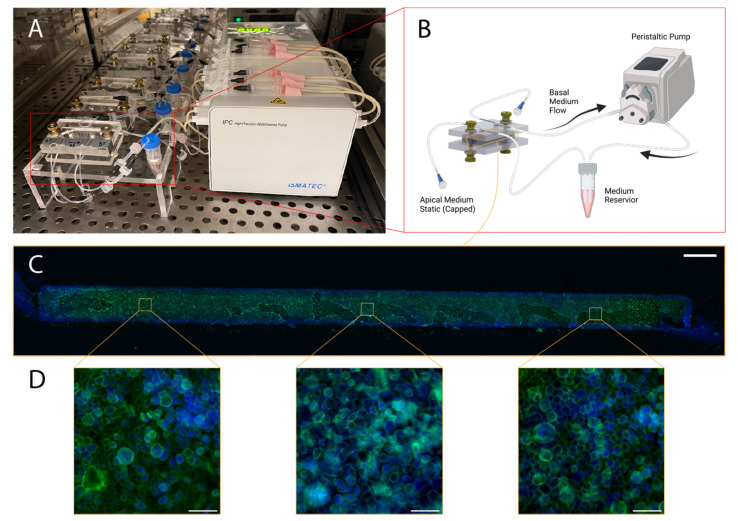
Illustration of airway-on-chip cell culturing protocol and representative results: (**A**) configuration of six chips in a cell-culture incubator with medium reservoirs and pumping set-up; (**B**) Schematic illustrating culture environment of single chip, with apical channel static and peristaltic-driven flow through basal channel (absent: luer-luer connections between microfluidic tubing and Ismatec 1.02 mm ID 2-stop peristaltic tubing), created with BioRender.com; (**C**) stitched microscopy image comprised of 10× magnification images capturing the entirety of a representative membrane post extraction and immunofluorescence staining (scale bar: 1.25 mm); green represents Alexa-fluor-488 Phalloidin, staining F-actin, while blue represents DAPI, present in the mounting medium. (**D**) three representative ROIs captured at 60× magnification along the length of the membrane (scale bar: 50 µm).

For the airway-on-chip, 71% of the 14 chips cultured with the methods described in Section 2.1, Section 2.2 and Section 2.3 produced extracted membranes with a confluent layer of airway-epithelia (depicted in Figure 10). Of these, all displayed the characteristic cobblestone-like morphology under F-actin immunofluorescence staining and were confined to the channel (as evidenced by the strict rectangular shape of the stained cells in Figure 9C). Consistent production of morphologically typical airway epithelial allows such in vitro models to be employed in applications such as aerosol inhalation studies, where the apical chamber medium can be replaced with air or aerosols of interest (for example cigarette smoke or diesel exhaust) [46,47].

## 4. Conclusions

PDMS-based organs-on-chip have enabled the development of next-generation in vitro cell culture models; this platform, however, does not come without its challenges. Leakage, flow system design and management, and PDMS bond strength are among the established difficulties associated with this platform that we have aimed to mitigate [22,48]. The flexibility offered by PDMS can be leveraged to quickly and inexpensively iterate on design and scale up production of more physiologically translatable in vitro models; limitations center largely on master mold production and fabrication success rates [49,50]. We have employed PDMS-focused 3D printing technologies that enable inexpensive (<CAD 5 in resin cost) and rapid (<2 h) mold production and described the improvements they offer over more conventional stereolithography 3D printers and resins due to PDMS cure-inhibition and surface roughness [17]. We have, further, described the inclusion of a number of fabrication strategies focused on improving robustness and fabrication success rate of PDMS organs-on-chip that utilize inexpensive, commercially available materials such as biopsy punches, copier transparency films, and needles.

We have applied subsets of these strategies to two different organs-on-chip and demonstrated the establishment of morphologically typical cell cultures through their application, achieving fabrication success rates of over 85% and average culture formation and maintenance success rates of approximately 65%. These strategies were focused on reducing the impact of persistent failure modes such as fluidic leakage (through optimizing the creation and reinforcement of fluidic ports) and increasing the ease of fluidics management and interfacing (through the use of luer-lock for every connection in the system). Employing liquid uncured PDMS as a sealant, either as a moat (blood–brain barrier chip) or as small volumes dispensed around top-loaded ports (airway-on-chip) reinforces needle and tubing connections while remaining resistant to chemical disinfectants (a limitation of epoxy-based reinforcement methods). Incorporation of luer connections simplifies fluidic changes (such as injection of medium for washing channels or in situ staining protocols) and channel clamping or blocking (essential to minimizing the risk of crossflow across porous membranes). We have also described methods to achieve uniform compression using inexpensive, commercially available fastening tools (thumb nuts, washers, and threaded rods) in conjunction with laser-cut cast acrylic (the single unit cost of which is under CAD 5 and required less than 10 min of processing time). This enables further reinforcement of PDMS-PDMS bonding as well as adds support in areas where PDMS is in contact with another material (such as the PET membrane used as a cell-culture substrate). We have found incorporation of compression improves control over cell proliferation and better confines growth to channel borders, essential for reproducible analysis.

Additional challenges associated with the use of PDMS in the organ-on-chip include the established tendency for PDMS to absorb small hydrophobic molecules [51]; there have been multiple strategies to combat this reported in the literature. These include the incorporation of coating solutions [52] as well as surface modifications [53]. While the strategies described in this article do not directly address these challenges, they can be used in conjunction with existing methods reported and cited above.

The methods described are a compromise between increasing the reproducibility of organs-on-chip fabrication and assembly, while providing straightforward procedures that allow for wider platform adoption by non-specialized users. Dependence on manual fabrication and assembly is less desirable, where variability can be introduced by the end user. However, more automated and standardized industrial processes can have larger overhead costs, may require complex protocols for specialized equipment, and may require users to spend a significant amount of time initially on device design, all of which can be prohibitive to the uptake and application of organs-on-chip for research. Fabrication tools such as 3D printers and laser cutters are becoming more common in research laboratories. The use of less time-consuming techniques with low-cost and ubiquitous consumables (such as the use of transparency films to improve PDMS flatness or using a PDMS moat for reinforcing the inlet and outlet ports), can be more easily and readily transferable to other applications for PDMS-based microfluidic devices and organ-on-chip development.

## Figures and Tables

**Table 1:** Key features for the airway-on-chip and the BBB chip.

Design Aspect	Airway-On-Chip	BBB Chip	Comments
PDMS Bonding	Partial cure	Oxygen plasma	Oxygen plasma treatment is the most commonly used option and is well characterized but requires an oxygen plasma cleaner [35]. Partial curing does not require additional hardware and has been shown in some reports to provide higher bond strength [22]. It, however, requires tight control over temperature for bond-strength reproducibility and may pose difficulties when manipulating more flexible and tacky partially cured PDMS layers.
I/O location	Top-loaded	Side-loaded	Side loaded fluidic ports are more amenable to dual-sided imaging
I/O port formation	Biopsy punch (0.5 mm OD)	Needle mold (0.5 mm OD)	Side loaded fluidics require sacrificial molding of ports, while top loading enables punching of I/O ports.
Fluidic seal reinforcement	PDMS seal + compressive clamp	PDMS encasement (“moat”)	See Section 2.2.2

**Table 2:** An overview of the checkpoints used to assess the outcome of different stages in the BBB chips timeline. These checkpoints include PDMS bonding, media perfusion, cell attachment and the formation of a confluent monolayer. Common and suspected failure modes are also described.

Description		Common and Suspected Failure Modes	
*Sample size of chips between checkpoints 1–2*		*17*	
Checkpoint 1	Successful PDMS-PDMS bonding based on complete transparency of PDMS pieces and manual leak test	100%	N/A
Checkpoint 2	Viable chips at the end of the experiment	88%	Tubing coming undone from needle on syringe that is dispensing media.
***Sample size of chips between checkpoints 3–4***		*8*	
Checkpoint 3	Chips with cell attachment	88%	Cell detachment due to uneven ECM coating, air bubbles in the channel, or insufficient media supply.
Checkpoint 4	Chips with a confluent monolayer	57%	Washing and fixing steps and disassembly of chips caused cells to lift before mounting membranes onto glass slides. Cell detachment due to uneven ECM coating.

## Data Availability

The data presented in this study are available on request from the corresponding author.

## Supplementary Materials

The following supporting information can be downloaded at:https://www.mdpi.com/article/10.3390/mi13101573/s1, Figure S1: Flowrate stability at 75 uL/min achieved with peristaltic (blue) and syringe (orange) pump against a programmed set-point (green). A ten-minute interval is expanded in an inset plot to better re-solve the periodicity of the profile midway through the approximately two-hour measurement; Figure S2: Flowrate stability at 15 uL/min with peristaltic (blue) and syringe (orange) pump against a pro-grammed set-point (green). An approximately 35-min-long interval is expanded to resolve the periodicity of the profile midway through the overnight measurement.
