# Supplementary material for: PDMS Organ-On-Chip Design and Fabrication: Strategies for Improving Fluidic Integration and Chip Robustness of Rapidly Prototyped Microfluidic In Vitro Models
Source: Micromachines (Basel). 2022 Sep 22;13(10):1573. doi: 10.3390/mi13101573 (PMC9609846; doi:10.3390/mi13101573)
Supplement: Supplementary file 1 [file micromachines-13-01573-s001.zip › micromachines-1874503-supplementary.pdf]

PDMS Organ-on-Chip Design and Fabrication: Strategies for Improving Fluidic Integration and Chip Robustness of Rapidly Prototyped Microfluidic In Vitro Models

Supplemental Materials

Methods and Materials

Master Mold Production

Parts made with the DLP printer used a proprietary resin purpose built for PDMS (Master Mold Resin, Creative CADworks, Vaughan, Canada) to produce 3D printed molds, and a computer-aided design (CAD) (Solidworks, CMC Microsystems) part was produced, and the standard triangle language (STL) file was imported to the Utility software (version 6.3.0.t3, Creative CadWorks) to convert the STL file to a .3DP file. The file was then uploaded to the DLP printer with a z-resolution selection of 30 μm, and the part was oriented to lay flat on the print bed, with the feature side facing upwards. After printing, the print was post-processed with two cycles of: rinsing in an isopropanol (IPA) (>95% purity) bath for 20 minutes, then air drying with compressed air. Then, the print was cured in an ultraviolet (UV) chamber (Formcure, Formlabs or CureZone, CadWorks) for 40-60 minutes at room temperature with the features side facing upwards and flipped over for further curing (20 minutes) with the feature side facing down. For printing on the SLA printer, the files were taken from the CAD software (Solidworks, CMC Microsystems) in STL format and uploaded onto the PreForm software (Version 3.12.2, FormLabs). A proprietary clear resin (Cat. no. RS-F2-GPCL-04, FormLabs) and a selected printing resolution of 25 μm were used. The parts were oriented on the build platform with a 45º angle and with as few supports on the feature side of the print as possible. Once printed, the parts were removed from the build platform and post-processed as per the manufacturer's recommendations. Briefly, the parts were added to an initial IPA bath for 5-10 minutes, then transferred to a second IPA bath for another 5 minutes. The parts were left to dry overnight in a fume hood and UV-cured on both sides for 30 minutes at 60ºC (FormCure, Formlabs).

Design of the Airway on Chip

The columnar features (considered punch-guides) serve multiple purposes; firstly, they ensure accurate positioning of the manual punch (particularly critical for the i/o ports corresponding to the basal channel, as this channel feature resides on bottom layer) and, secondly, they decrease the likelihood of deformed or slanted manual punching. The airway-on-chip utilizes the PDMS partial-curing method of bonding (further discussed in a later section); as such, punching and alignment of the two layers occurs before the PDMS has reached its final stiffness, which can complicate the manual punching process. The punch guides penetrate 3mm into the 6-mm-deep layer; any further than this depth was found to markedly increase the frequency of feature damage or fracture during the demolding process, due to the torque applied to the high-aspect-ratio posts when lifting the PDMS along the mold edges.

Characterization of Flow Rate Stability

Both syringe and peristaltic pumps were employed to drive flow of culture medium through the organs-on-chip during the course of experiments. Pump type was generally chosen based on the desired flow rates. In order to obtain a more direct comparison of pump performance, we employed a microfluidic flow sensor (Fluigent, FLOW UNIT M, Cat. FLU-M-D) in line with both the syringe (KD Scientific, Cat: 78-0230; used with 10 mL Syringe, Becton Dickinson and Company) and peristaltic (Ismatec, Cat: 78000-31) pump

employed during organ-on-chip cell culture to characterize the temporal profile of flowrate under flow of ultrapure water. Due to volume constraints as well as sensor working range limits, two measurements are provided; Figure S1 exhibits a 75 uL/min set point measured over a duration of approximately two hours, while Figure S2 exhibits a 15 uL/min set point measured overnight (approximately 10.5 hours). The measurement dispersion, as expected, is considerably higher when flow is peristaltic-pump driven, though periodicity about the set-point is also evident with syringe-pump driven flow. The percentage coefficient of variation for syringe and peristaltic driven flow at 15 uL/min is 4% and 29%, respectively, while at 75 uL/min 4% and 20% respectively.

These data may be indicative of a syringe pump being more applicable in cases of where lower and more stable flow rates are required. Accordingly, in the reported experiments, a syringe pump was used at a set-point of 1 uL/min while a peristaltic pump was used in ranges of 300-540 uL/min. As, between the two flow rates measured, the coefficient of variation (CV) appears to decrease with higher flowrate, the percentage error obtained at the 75 uL/min flow rate (20%) may be used to establish an approximate and conservative error on the shear-stress obtained during airway-on-chip experiments (which employed a flow rate higher than those characterized in Figures 1-2 and, if the trend identified above holds, may be associated with lower relative flow rate fluctuation). At 300 uL/min, then, the shear stress exerted basally is 0.363 $\pm$ 0.073 dyne/cm^2, while at 540 uL/min the shear stress exerted basally is 0.653 $\pm$ 0.131 dyne/cm^2.

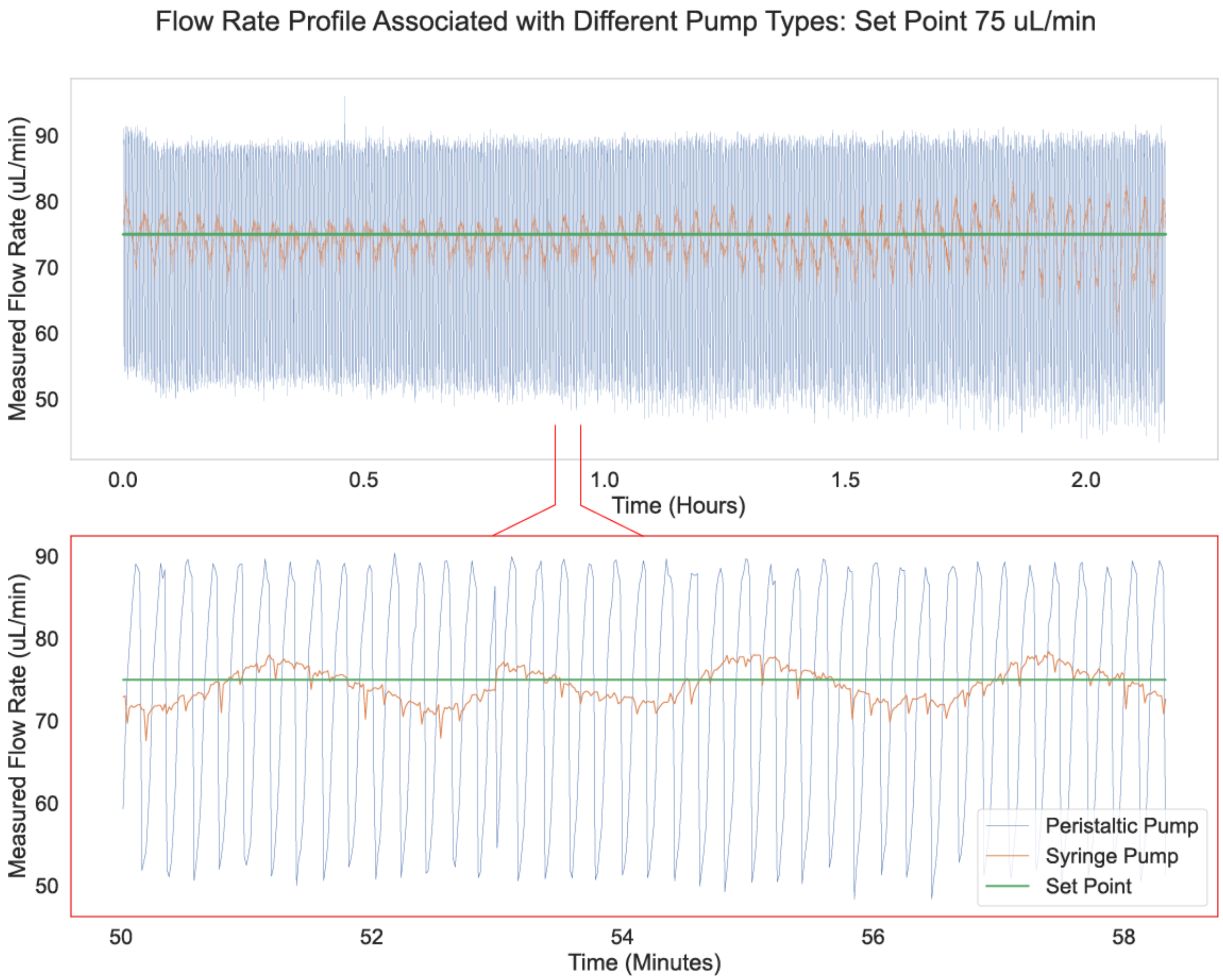

Figure S1. Flowrate stability at 75 uL/min achieved with peristaltic (blue) and syringe (orange) pump against a programmed set-point (green). A ten-minute interval is expanded in an inset plot to better resolve the periodicity of the profile midway through the approximately two-hour measurement.

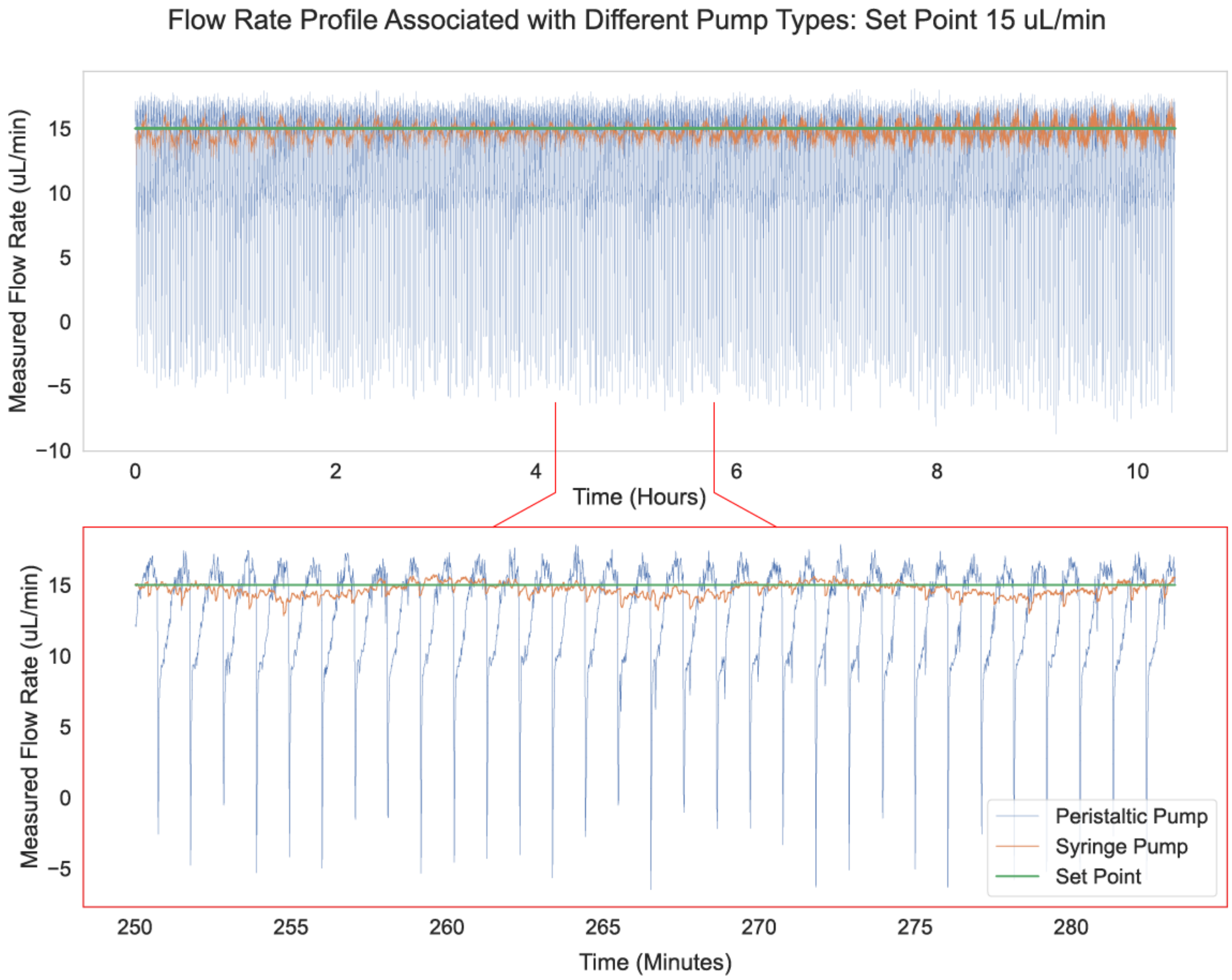

Figure S2. Flowrate stability at 15 uL/min with peristaltic (blue) and syringe (orange) pump against a programmed set-point (green). An approximately 35-minute-long interval is expanded to resolve the periodicity of the profile midway through the overnight measurement. .
